# [*N*-(3-Meth­oxy-2-oxidobenzyl­idene-κ*O* ^2^)alaninato-κ^2^ *N*,*O*]diphenyl­tin(IV)

**DOI:** 10.1107/S1600536809002426

**Published:** 2009-01-28

**Authors:** Hong-Jun Yang, Yan-Qiu Dang

**Affiliations:** a Research Center for Eco-Environmental Sciences of the Yellow River Delta, Binzhou University, Binzhou 256600, People’s Republic of China; b Department of Chemistry and Chemical Engineering, Binzhou University, Binzhou 256600, People’s Republic of China

## Abstract

The Sn atom of the title compound, [Sn(C_6_H_5_)_2_(C_11_H_11_NO_4_)], adopts a distorted SnNC_2_O_2_ trigonal–bipyramidal geometry with the O atoms in the axial positions. The metal atom forms five- and six-membered chelate rings with the *O*,*N*,*O*′-tridentate ligand.

## Related literature

For background, see: Rivera *et al.* (2006 ▶). For related structures, see: Beltran *et al.* (2003 ▶); Tian *et al.* (2007 ▶).
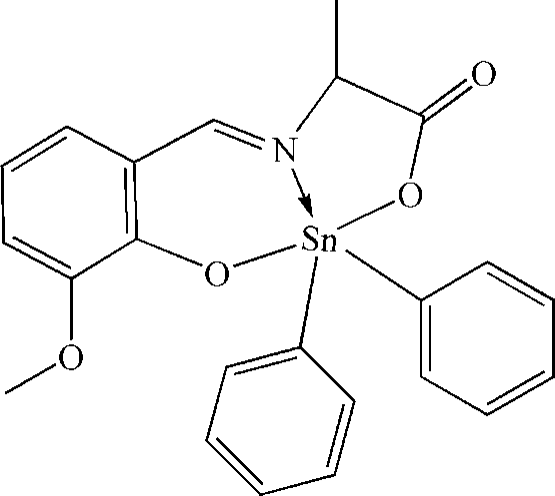


## Experimental

### 

#### Crystal data

[Sn(C_6_H_5_)_2_(C_11_H_11_NO_4_)]
*M* *_r_* = 494.10
Monoclinic, 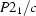

*a* = 13.3881 (10) Å
*b* = 9.0304 (6) Å
*c* = 17.3398 (12) Å
β = 95.077 (1)°
*V* = 2088.2 (3) Å^3^
*Z* = 4
Mo *K*α radiation
μ = 1.25 mm^−1^
*T* = 295 (2) K
0.16 × 0.12 × 0.10 mm

#### Data collection

Bruker APEX area-detector diffractometer
Absorption correction: multi-scan (*SADABS*; Bruker, 2002 ▶) *T*_min_ = 0.825, *T*_max_ = 0.885
16481 measured reflections
4326 independent reflections
3475 reflections with *I* > 2σ(*I*)
*R*_int_ = 0.022

#### Refinement

*R*[*F* ^2^ > 2σ(*F* ^2^)] = 0.024
*wR*(*F* ^2^) = 0.063
*S* = 1.04
4326 reflections
262 parameters
H-atom parameters constrained
Δρ_max_ = 0.44 e Å^−3^
Δρ_min_ = −0.26 e Å^−3^

### 

Data collection: *SMART* (Bruker, 2002 ▶); cell refinement: *SAINT* (Bruker, 2002 ▶); data reduction: *SAINT*; program(s) used to solve structure: *SHELXS97* (Sheldrick, 2008 ▶); program(s) used to refine structure: *SHELXL97* (Sheldrick, 2008 ▶); molecular graphics: *ORTEP-3 for Windows* (Farrugia, 1997 ▶); software used to prepare material for publication: *SHELXL97*.

## Supplementary Material

Crystal structure: contains datablocks global, I. DOI: 10.1107/S1600536809002426/hb2900sup1.cif

Structure factors: contains datablocks I. DOI: 10.1107/S1600536809002426/hb2900Isup2.hkl

Additional supplementary materials:  crystallographic information; 3D view; checkCIF report

## Figures and Tables

**Table 1:** Selected bond lengths (Å)

Sn1—C1	2.109 (2)
Sn1—C7	2.113 (2)
Sn1—O1	2.1360 (16)
Sn1—O3	2.0752 (14)
Sn1—N1	2.1493 (17)

**Fig. 1.**
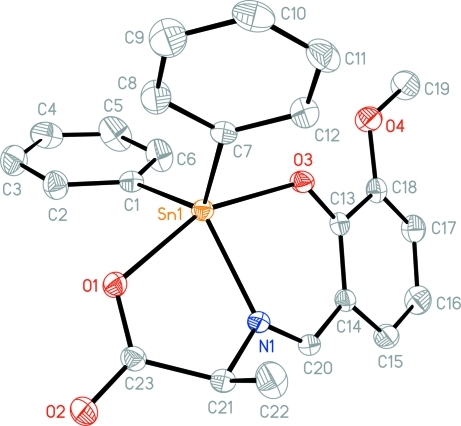
The structure of (I) with displacement ellipsoids drawn at the 30% probability level. The H atoms have been omitted for clarity.

## supplementary crystallographic information

### Comment

The diorganotin complexes with Schiff bases derived from α-amino acids continue to receive attention because of their biological activities and their quadratic nonlinear optical properties (*e.g.* Rivera *et al.*, 2006). The structures of two diorganotin complexes based on the Schiff base ligand [*N*-(2-hydroxyphenylmethylene)alanine, [*N*-(2-oxidophenylmethylene)alaninato]diphenyltin(IV) (Beltran *et al.*, 2003) and dicyclohexyl[*N*-(3,5-dibromo-2-oxidophenylmethylene)alaninato]tin(IV) (Tian *et al.*, 2007) have been reported. As a continuation of these studies, the structure of the title compound, (I), is now described.

The coordination geometry of the Sn atom in (I) is that of a distorted trigonal bipyramid with two phenyl groups and the imino N1 atom occupying the equatorial positions and the axial positions being occupied by a unidentate carboxylate O1 atom and phenoxide O3 atom (Fig. 1). The Sn atom is 0.061 (3) Å out of the NC2 trigonal plane in the direction of the O3 atom. The bond length of Sn1—O1 [2.1360 (16) Å] is longer than that of Sn1—O3 [2.0752 (14) Å]. The bond angle O1—Sn1—O3 is 158.35 (6)°, which is slightly larger than that observed in [*N*-(2-oxidophenylmethylene)alaninato]diphenyltin(IV) [156.90 (9)°] (Beltran *et al.*, 2003) and dicyclohexyl[*N*-(3,5-dibromo-2-oxidophenylmethylene)alaninato]tin(IV) [154.9 (1)°] (Tian *et al.*, 2007). The monodentate mode of coordination of carboxylate is reflected in the disparate C23—O1 and C23—O2 bond lengths of 1.290 (3) and 1.213 (3) Å, respectively.

### Experimental

The title compound was prepared by the reaction of diphenyltin dichloride (0.69 g, 2 mmol) with potassium *N*-(3-methoxy-2-hydroxyphenylmethylene)alaninate (0.29 g, 2 mmol) in the presence of Et_3_N (0.20 g, 2 mmol) in methanol (30 ml). The reaction mixture was refluxed for 2 h and filtered. The yellow solid obtained by removal of solvent under reduced pressure was recrystallized from methanol and yellow blocks of (I) were obtained from dichloromethane–hexane (1:1 v/v) by slow evaporation at room temperature (yield 76%, m.p. 535–536 K).

### Refinement

The H atoms were placed at calculated positions (C—H = 0.93–0.98Å) and refined as riding with *U*_iso_(H) = 1.2U_eq_(C) or 1.5U_eq_(methyl C).

### Crystal data

**Table:**

[Sn(C_6_H_5_)_2_(C_11_H_11_NO_4_)]	*F*(000) = 992
*M**_r_* = 494.10	*D*_x_ = 1.572 Mg m^−^^3^
Monoclinic, *P*2_1_/*c*	Mo *K*α radiation, λ = 0.71073 Å
Hall symbol: -P 2ybc	Cell parameters from 6772 reflections
*a* = 13.3881 (10) Å	θ = 2.4–27.7°
*b* = 9.0304 (6) Å	µ = 1.25 mm^−^^1^
*c* = 17.3398 (12) Å	*T* = 295 K
β = 95.077 (1)°	Block, yellow
*V* = 2088.2 (3) Å^3^	0.16 × 0.12 × 0.10 mm
*Z* = 4	

### Data collection

**Table:**

Bruker SMART APEX area-detector diffractometer	4326 independent reflections
Radiation source: fine-focus sealed tube	3475 reflections with *I* > 2σ(*I*)
graphite	*R*_int_ = 0.022
φ and ω scans	θ_max_ = 26.5°, θ_min_ = 1.5°
Absorption correction: multi-scan (*SADABS*; Bruker, 2002)	*h* = −16→16
*T*_min_ = 0.825, *T*_max_ = 0.885	*k* = −11→11
16481 measured reflections	*l* = −21→20

### Refinement

**Table:**

Refinement on *F*^2^	Primary atom site location: structure-invariant direct methods
Least-squares matrix: full	Secondary atom site location: difference Fourier map
*R*[*F*^2^ > 2σ(*F*^2^)] = 0.024	Hydrogen site location: inferred from neighbouring sites
*wR*(*F*^2^) = 0.063	H-atom parameters constrained
*S* = 1.04	*w* = 1/[σ^2^(*F*_o_^2^) + (0.029*P*)^2^ + 0.7005*P*] where *P* = (*F*_o_^2^ + 2*F*_c_^2^)/3
4326 reflections	(Δ/σ)_max_ = 0.002
262 parameters	Δρ_max_ = 0.44 e Å^−^^3^
0 restraints	Δρ_min_ = −0.26 e Å^−^^3^

### Special details

**Table:**

Geometry. All e.s.d.'s (except the e.s.d. in the dihedral angle between two l.s. planes) are estimated using the full covariance matrix. The cell e.s.d.'s are taken into account individually in the estimation of e.s.d.'s in distances, angles and torsion angles; correlations between e.s.d.'s in cell parameters are only used when they are defined by crystal symmetry. An approximate (isotropic) treatment of cell e.s.d.'s is used for estimating e.s.d.'s involving l.s. planes.
Refinement. Refinement of *F*^2^ against ALL reflections. The weighted *R*-factor *wR* and goodness of fit *S* are based on *F*^2^, conventional *R*-factors *R* are based on *F*, with *F* set to zero for negative *F*^2^. The threshold expression of *F*^2^ > σ(*F*^2^) is used only for calculating *R*-factors(gt) *etc*. and is not relevant to the choice of reflections for refinement. *R*-factors based on *F*^2^ are statistically about twice as large as those based on *F*, and *R*- factors based on ALL data will be even larger.

### Fractional atomic coordinates and isotropic or equivalent isotropic displacement parameters (Å^2^)

**Table:**

	*x*	*y*	*z*	*U*_iso_*/*U*_eq_	
Sn1	0.292357 (11)	0.503657 (15)	0.348838 (8)	0.03542 (6)	
N1	0.32598 (13)	0.28485 (19)	0.30979 (10)	0.0385 (4)	
O1	0.41792 (13)	0.53434 (17)	0.28262 (10)	0.0467 (4)	
O2	0.52345 (14)	0.4293 (2)	0.20800 (11)	0.0638 (5)	
O3	0.18288 (11)	0.39202 (16)	0.40344 (9)	0.0450 (4)	
O4	0.07445 (14)	0.3448 (2)	0.51854 (10)	0.0609 (5)	
C1	0.36449 (17)	0.6022 (2)	0.44906 (13)	0.0415 (5)	
C2	0.4416 (2)	0.7013 (3)	0.44224 (16)	0.0575 (7)	
H2	0.4664	0.7151	0.3943	0.069*	
C3	0.4825 (2)	0.7803 (4)	0.50548 (18)	0.0727 (9)	
H3	0.5340	0.8476	0.5001	0.087*	
C4	0.4471 (2)	0.7590 (4)	0.57586 (18)	0.0782 (10)	
H4	0.4736	0.8131	0.6185	0.094*	
C5	0.3736 (3)	0.6596 (5)	0.58371 (17)	0.0905 (11)	
H5	0.3515	0.6432	0.6323	0.109*	
C6	0.3305 (2)	0.5816 (4)	0.52079 (15)	0.0706 (8)	
H6	0.2787	0.5154	0.5269	0.085*	
C7	0.18958 (16)	0.6179 (2)	0.27068 (12)	0.0403 (5)	
C8	0.2149 (2)	0.7542 (3)	0.24244 (18)	0.0664 (8)	
H8	0.2765	0.7965	0.2588	0.080*	
C9	0.1487 (3)	0.8287 (4)	0.1896 (2)	0.0847 (10)	
H9	0.1664	0.9206	0.1708	0.102*	
C10	0.0582 (2)	0.7683 (4)	0.16505 (18)	0.0717 (9)	
H10	0.0144	0.8184	0.1295	0.086*	
C11	0.0321 (2)	0.6336 (4)	0.19299 (16)	0.0660 (8)	
H11	−0.0297	0.5920	0.1766	0.079*	
C12	0.0976 (2)	0.5588 (3)	0.24582 (16)	0.0554 (6)	
H12	0.0791	0.4674	0.2647	0.066*	
C13	0.18852 (16)	0.2585 (2)	0.43474 (13)	0.0383 (5)	
C14	0.24719 (17)	0.1438 (3)	0.40775 (13)	0.0426 (5)	
C15	0.2462 (2)	0.0026 (3)	0.44269 (19)	0.0591 (7)	
H15	0.2848	−0.0735	0.4247	0.071*	
C16	0.1894 (2)	−0.0228 (3)	0.50210 (19)	0.0670 (8)	
H16	0.1900	−0.1159	0.5251	0.080*	
C17	0.1301 (2)	0.0894 (3)	0.52905 (15)	0.0575 (7)	
H17	0.0906	0.0702	0.5694	0.069*	
C18	0.12930 (18)	0.2276 (3)	0.49671 (13)	0.0461 (5)	
C19	0.0072 (2)	0.3215 (4)	0.57615 (18)	0.0810 (10)	
H19A	−0.0259	0.4129	0.5862	0.121*	
H19B	0.0438	0.2872	0.6228	0.121*	
H19C	−0.0417	0.2487	0.5584	0.121*	
C20	0.30727 (16)	0.1628 (2)	0.34391 (13)	0.0434 (5)	
H20	0.3358	0.0776	0.3252	0.052*	
C21	0.38800 (17)	0.2828 (3)	0.24357 (13)	0.0441 (5)	
H21	0.4326	0.1967	0.2476	0.053*	
C22	0.3194 (2)	0.2741 (4)	0.16856 (15)	0.0698 (8)	
H22A	0.2807	0.1846	0.1681	0.105*	
H22B	0.3592	0.2745	0.1252	0.105*	
H22C	0.2751	0.3579	0.1652	0.105*	
C23	0.45012 (17)	0.4244 (3)	0.24431 (13)	0.0422 (5)	

### Atomic displacement parameters (Å^2^)

**Table:**

	*U*^11^	*U*^22^	*U*^33^	*U*^12^	*U*^13^	*U*^23^
Sn1	0.03617 (10)	0.03374 (10)	0.03691 (10)	0.00146 (6)	0.00633 (6)	−0.00038 (6)
N1	0.0354 (10)	0.0370 (10)	0.0442 (10)	−0.0001 (8)	0.0092 (8)	−0.0041 (8)
O1	0.0492 (10)	0.0413 (9)	0.0520 (10)	−0.0067 (7)	0.0170 (8)	−0.0066 (7)
O2	0.0622 (12)	0.0598 (11)	0.0751 (13)	−0.0118 (10)	0.0371 (10)	−0.0114 (10)
O3	0.0425 (9)	0.0395 (9)	0.0553 (10)	0.0031 (7)	0.0178 (7)	0.0053 (7)
O4	0.0676 (12)	0.0582 (11)	0.0616 (11)	−0.0035 (9)	0.0319 (9)	−0.0034 (9)
C1	0.0420 (12)	0.0427 (12)	0.0393 (12)	0.0061 (10)	0.0013 (10)	−0.0014 (10)
C2	0.0552 (16)	0.0639 (17)	0.0529 (15)	−0.0079 (13)	0.0025 (12)	−0.0048 (13)
C3	0.0643 (19)	0.076 (2)	0.075 (2)	−0.0132 (16)	−0.0058 (16)	−0.0190 (16)
C4	0.065 (2)	0.100 (3)	0.066 (2)	0.0131 (18)	−0.0173 (16)	−0.0356 (18)
C5	0.081 (2)	0.148 (3)	0.0427 (17)	−0.009 (2)	0.0074 (15)	−0.0224 (19)
C6	0.0678 (19)	0.099 (2)	0.0462 (16)	−0.0162 (18)	0.0093 (14)	−0.0058 (16)
C7	0.0389 (12)	0.0427 (12)	0.0398 (12)	0.0056 (10)	0.0064 (10)	−0.0013 (10)
C8	0.0541 (16)	0.0578 (17)	0.085 (2)	−0.0018 (13)	−0.0081 (14)	0.0189 (15)
C9	0.078 (2)	0.069 (2)	0.105 (3)	0.0064 (17)	−0.0068 (19)	0.0410 (18)
C10	0.0600 (19)	0.084 (2)	0.069 (2)	0.0265 (17)	−0.0059 (15)	0.0106 (17)
C11	0.0445 (15)	0.084 (2)	0.0676 (18)	0.0065 (14)	−0.0082 (13)	−0.0043 (16)
C12	0.0507 (15)	0.0580 (15)	0.0571 (16)	−0.0025 (13)	0.0030 (12)	0.0009 (13)
C13	0.0336 (11)	0.0410 (12)	0.0399 (12)	−0.0044 (9)	0.0006 (9)	0.0014 (9)
C14	0.0359 (12)	0.0401 (12)	0.0520 (14)	−0.0024 (10)	0.0040 (10)	0.0032 (10)
C15	0.0550 (16)	0.0432 (15)	0.080 (2)	0.0048 (11)	0.0086 (15)	0.0113 (13)
C16	0.072 (2)	0.0533 (17)	0.076 (2)	−0.0052 (14)	0.0070 (17)	0.0236 (14)
C17	0.0606 (16)	0.0632 (17)	0.0498 (15)	−0.0092 (14)	0.0106 (13)	0.0099 (13)
C18	0.0444 (13)	0.0507 (14)	0.0434 (13)	−0.0088 (11)	0.0041 (10)	−0.0001 (11)
C19	0.084 (2)	0.085 (2)	0.081 (2)	−0.0113 (18)	0.0471 (18)	−0.0079 (18)
C20	0.0364 (12)	0.0348 (12)	0.0594 (15)	0.0014 (9)	0.0062 (11)	−0.0048 (10)
C21	0.0411 (12)	0.0447 (13)	0.0476 (13)	0.0002 (10)	0.0110 (10)	−0.0062 (10)
C22	0.0623 (18)	0.098 (2)	0.0494 (16)	−0.0171 (16)	0.0049 (13)	−0.0207 (15)
C23	0.0406 (12)	0.0460 (13)	0.0406 (12)	−0.0002 (10)	0.0065 (10)	−0.0008 (10)

### Geometric parameters (Å, °)

**Table:**

Sn1—C1	2.109 (2)	C9—C10	1.363 (4)
Sn1—C7	2.113 (2)	C9—H9	0.9300
Sn1—O1	2.1360 (16)	C10—C11	1.366 (4)
Sn1—O3	2.0752 (14)	C10—H10	0.9300
Sn1—N1	2.1493 (17)	C11—C12	1.387 (4)
N1—C20	1.286 (3)	C11—H11	0.9300
N1—C21	1.475 (3)	C12—H12	0.9300
O1—C23	1.290 (3)	C13—C14	1.405 (3)
O2—C23	1.213 (3)	C13—C18	1.418 (3)
O3—C13	1.321 (2)	C14—C15	1.412 (3)
O4—C18	1.361 (3)	C14—C20	1.435 (3)
O4—C19	1.418 (3)	C15—C16	1.353 (4)
C1—C6	1.374 (3)	C15—H15	0.9300
C1—C2	1.379 (3)	C16—C17	1.394 (4)
C2—C3	1.380 (4)	C16—H16	0.9300
C2—H2	0.9300	C17—C18	1.368 (3)
C3—C4	1.361 (4)	C17—H17	0.9300
C3—H3	0.9300	C19—H19A	0.9600
C4—C5	1.348 (5)	C19—H19B	0.9600
C4—H4	0.9300	C19—H19C	0.9600
C5—C6	1.381 (4)	C20—H20	0.9300
C5—H5	0.9300	C21—C23	1.525 (3)
C6—H6	0.9300	C21—C22	1.527 (3)
C7—C12	1.376 (3)	C21—H21	0.9800
C7—C8	1.378 (3)	C22—H22A	0.9600
C8—C9	1.391 (4)	C22—H22B	0.9600
C8—H8	0.9300	C22—H22C	0.9600
			
O3—Sn1—C1	96.86 (8)	C10—C11—H11	120.0
O3—Sn1—C7	94.80 (7)	C12—C11—H11	120.0
C1—Sn1—C7	123.18 (9)	C7—C12—C11	121.0 (3)
O3—Sn1—O1	158.35 (6)	C7—C12—H12	119.5
C1—Sn1—O1	93.64 (8)	C11—C12—H12	119.5
C7—Sn1—O1	95.20 (8)	O3—C13—C14	123.3 (2)
O3—Sn1—N1	82.71 (6)	O3—C13—C18	118.4 (2)
C1—Sn1—N1	123.66 (8)	C14—C13—C18	118.3 (2)
C7—Sn1—N1	112.92 (8)	C13—C14—C15	119.7 (2)
O1—Sn1—N1	75.71 (6)	C13—C14—C20	122.5 (2)
C20—N1—C21	119.56 (19)	C15—C14—C20	117.8 (2)
C20—N1—Sn1	126.04 (15)	C16—C15—C14	120.5 (3)
C21—N1—Sn1	113.85 (14)	C16—C15—H15	119.8
C23—O1—Sn1	119.47 (15)	C14—C15—H15	119.8
C13—O3—Sn1	127.85 (13)	C15—C16—C17	120.5 (2)
C18—O4—C19	118.2 (2)	C15—C16—H16	119.8
C6—C1—C2	118.4 (2)	C17—C16—H16	119.8
C6—C1—Sn1	121.65 (19)	C18—C17—C16	120.7 (3)
C2—C1—Sn1	119.66 (17)	C18—C17—H17	119.7
C1—C2—C3	121.0 (3)	C16—C17—H17	119.7
C1—C2—H2	119.5	O4—C18—C17	125.5 (2)
C3—C2—H2	119.5	O4—C18—C13	114.1 (2)
C4—C3—C2	119.6 (3)	C17—C18—C13	120.4 (2)
C4—C3—H3	120.2	O4—C19—H19A	109.5
C2—C3—H3	120.2	O4—C19—H19B	109.5
C5—C4—C3	120.0 (3)	H19A—C19—H19B	109.5
C5—C4—H4	120.0	O4—C19—H19C	109.5
C3—C4—H4	120.0	H19A—C19—H19C	109.5
C4—C5—C6	121.1 (3)	H19B—C19—H19C	109.5
C4—C5—H5	119.4	N1—C20—C14	127.0 (2)
C6—C5—H5	119.4	N1—C20—H20	116.5
C1—C6—C5	119.9 (3)	C14—C20—H20	116.5
C1—C6—H6	120.1	N1—C21—C23	109.11 (18)
C5—C6—H6	120.1	N1—C21—C22	109.03 (19)
C12—C7—C8	118.5 (2)	C23—C21—C22	109.7 (2)
C12—C7—Sn1	121.71 (18)	N1—C21—H21	109.7
C8—C7—Sn1	119.80 (18)	C23—C21—H21	109.7
C7—C8—C9	120.2 (3)	C22—C21—H21	109.7
C7—C8—H8	119.9	C21—C22—H22A	109.5
C9—C8—H8	119.9	C21—C22—H22B	109.5
C10—C9—C8	120.6 (3)	H22A—C22—H22B	109.5
C10—C9—H9	119.7	C21—C22—H22C	109.5
C8—C9—H9	119.7	H22A—C22—H22C	109.5
C9—C10—C11	119.6 (3)	H22B—C22—H22C	109.5
C9—C10—H10	120.2	O2—C23—O1	124.2 (2)
C11—C10—H10	120.2	O2—C23—C21	119.5 (2)
C10—C11—C12	120.1 (3)	O1—C23—C21	116.3 (2)
			
O3—Sn1—N1—C20	−25.19 (18)	C12—C7—C8—C9	−0.5 (4)
C1—Sn1—N1—C20	68.2 (2)	Sn1—C7—C8—C9	178.5 (2)
C7—Sn1—N1—C20	−117.34 (19)	C7—C8—C9—C10	0.0 (5)
O1—Sn1—N1—C20	153.0 (2)	C8—C9—C10—C11	0.4 (5)
O3—Sn1—N1—C21	163.36 (15)	C9—C10—C11—C12	−0.2 (5)
C1—Sn1—N1—C21	−103.22 (16)	C8—C7—C12—C11	0.6 (4)
C7—Sn1—N1—C21	71.22 (16)	Sn1—C7—C12—C11	−178.3 (2)
O1—Sn1—N1—C21	−18.44 (14)	C10—C11—C12—C7	−0.3 (4)
O3—Sn1—O1—C23	11.9 (3)	Sn1—O3—C13—C14	−30.4 (3)
C1—Sn1—O1—C23	130.87 (18)	Sn1—O3—C13—C18	152.16 (16)
C7—Sn1—O1—C23	−105.33 (18)	O3—C13—C14—C15	−177.7 (2)
N1—Sn1—O1—C23	7.03 (16)	C18—C13—C14—C15	−0.3 (3)
C1—Sn1—O3—C13	−87.66 (18)	O3—C13—C14—C20	0.7 (3)
C7—Sn1—O3—C13	148.07 (18)	C18—C13—C14—C20	178.1 (2)
O1—Sn1—O3—C13	30.8 (3)	C13—C14—C15—C16	−0.2 (4)
N1—Sn1—O3—C13	35.53 (18)	C20—C14—C15—C16	−178.7 (3)
O3—Sn1—C1—C6	−1.4 (2)	C14—C15—C16—C17	0.8 (5)
C7—Sn1—C1—C6	98.9 (2)	C15—C16—C17—C18	−0.9 (5)
O1—Sn1—C1—C6	−162.4 (2)	C19—O4—C18—C17	−4.3 (4)
N1—Sn1—C1—C6	−87.2 (2)	C19—O4—C18—C13	175.4 (2)
O3—Sn1—C1—C2	−174.98 (19)	C16—C17—C18—O4	−179.9 (3)
C7—Sn1—C1—C2	−74.7 (2)	C16—C17—C18—C13	0.4 (4)
O1—Sn1—C1—C2	24.0 (2)	O3—C13—C18—O4	−2.0 (3)
N1—Sn1—C1—C2	99.2 (2)	C14—C13—C18—O4	−179.5 (2)
C6—C1—C2—C3	−1.3 (4)	O3—C13—C18—C17	177.7 (2)
Sn1—C1—C2—C3	172.5 (2)	C14—C13—C18—C17	0.2 (3)
C1—C2—C3—C4	0.7 (5)	C21—N1—C20—C14	−178.7 (2)
C2—C3—C4—C5	1.1 (5)	Sn1—N1—C20—C14	10.3 (3)
C3—C4—C5—C6	−2.3 (6)	C13—C14—C20—N1	9.1 (4)
C2—C1—C6—C5	0.1 (5)	C15—C14—C20—N1	−172.4 (2)
Sn1—C1—C6—C5	−173.6 (3)	C20—N1—C21—C23	−146.0 (2)
C4—C5—C6—C1	1.7 (6)	Sn1—N1—C21—C23	26.0 (2)
O3—Sn1—C7—C12	−29.5 (2)	C20—N1—C21—C22	94.2 (3)
C1—Sn1—C7—C12	−130.88 (19)	Sn1—N1—C21—C22	−93.8 (2)
O1—Sn1—C7—C12	131.3 (2)	Sn1—O1—C23—O2	−176.5 (2)
N1—Sn1—C7—C12	54.6 (2)	Sn1—O1—C23—C21	5.7 (3)
O3—Sn1—C7—C8	151.6 (2)	N1—C21—C23—O2	161.4 (2)
C1—Sn1—C7—C8	50.2 (2)	C22—C21—C23—O2	−79.3 (3)
O1—Sn1—C7—C8	−47.6 (2)	N1—C21—C23—O1	−20.8 (3)
N1—Sn1—C7—C8	−124.3 (2)	C22—C21—C23—O1	98.6 (2)
